# Comparative Molecular Dynamics Simulations Provide Insight Into Antibiotic Interactions: A Case Study Using the Enzyme L,L-Diaminopimelate Aminotransferase (DapL)

**DOI:** 10.3389/fmolb.2020.00046

**Published:** 2020-03-24

**Authors:** Lily E. Adams, Patrick Rynkiewicz, Gregory A. Babbitt, Jamie S. Mortensen, Rachel A. North, Renwick C. J. Dobson, André O. Hudson

**Affiliations:** ^1^Thomas H. Gosnell School of Life Sciences, Rochester Institute of Technology, Rochester, NY, United States; ^2^Department of Biomedical Engineering, Rochester Institute of Technology, Rochester, NY, United States; ^3^Biomolecular Interaction Centre and School of Biological Sciences, University of Canterbury, Christchurch, New Zealand; ^4^Department of Biochemistry and Molecular Biology, Bio21 Molecular Science and Biotechnology Institute, University of Melbourne, Melbourne, VIC, Australia

**Keywords:** L,L-diaminopimelate aminotransferase, molecular dynamics, ligand binding, antibiotics, L-lysine, diaminopimelate, peptidoglycan

## Abstract

The L,L-diaminopimelate aminotransferase (DapL) pathway, a recently discovered variant of the lysine biosynthetic pathway, is an attractive pipeline to identify targets for the development of novel antibiotic compounds. DapL is a homodimer that catalyzes the conversion of tetrahydrodipicolinate to L,L-diaminopimelate in a single transamination reaction. The penultimate and ultimate products of the lysine biosynthesis pathway, *meso*-diaminopimelate and lysine, are key components of the Gram-negative and Gram-positive bacterial peptidoglycan cell wall. Humans are not able to synthesize lysine, and DapL has been identified in 13% of bacteria whose genomes have been sequenced and annotated to date, thus it is an attractive target for the development of narrow spectrum antibiotics through the prevention of both lysine biosynthesis and peptidoglycan crosslinking. To address the common lack of structural information when conducting compound screening experiments and provide support for the use of modeled structures, our analyses utilized inferred structures from related homologous enzymes. Using a comprehensive and comparative molecular dynamics (MD) software package—DROIDS (Detecting Relative Outlier Impacts in Dynamic Simulations) 2.0, we investigated the binding dynamics of four previously identified antagonistic ligands of DapL from *Verrucomicrobium spinosum*, a non-pathogenic relative of *Chlamydia trachomatis*. Here, we present putative docking positions of the four ligands and provide confirmatory comparative molecular dynamics simulations supporting the conformations. The simulations performed in this study can be applied to evaluate putative targets to predict compound effectiveness prior to *in vivo* and *in vitro* experimentation. Moreover, this approach has the potential to streamline the process of antibiotic development.

## Introduction

**Figure 1:**
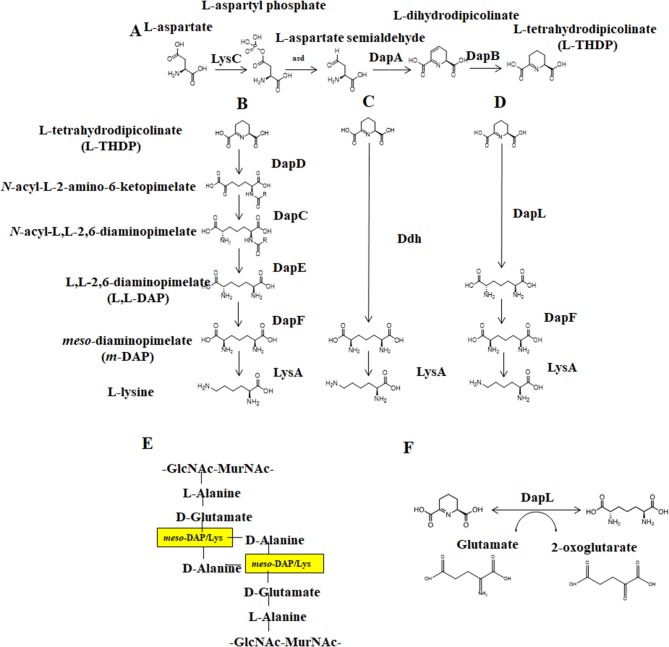
The Diaminopimelate pathways for lysine biosynthesis. **(A)** The synthesis of THDP from aspartate, **(B)** The acyl pathways, **(C)** the Ddh pathway, **(D)** the DapL pathway **(E)** Peptidoglycan crosslinking between disaccharide layers, GlcNAc-MurNAc, as mediated by meso-diaminopimelate/lysine and **(F)** The reversible transamination reaction catalyzed by DapL. DapC, acyl-diaminopimelate aminotransferase; DapE, acyl-diaminopimelate deacylase; DapD, 2,3,4,5-tetrahydropyridine-2,6-dicarboxylate-N-acyltransferase; DapF, acyl-diaminopimelate deacylase, DapL, L,L-diaminopimelate aminotransferase; Ddh, diaminopimelate dehydrogenase; LysA, diaminopimelate decarboxylase.

Novel targets and approaches to facilitate the development/discovery of antibiotics are highly sought after to combat the rise of single and multidrug resistant bacterial infections (Ventola, 2015). Bacterial enzymes that are involved in the anabolism of the nine nutritionally essential proteogenic amino acids (histidine, isoleucine, leucine, methionine, phenylalanine, threonine, tryptophan, valine, and lysine) are among the attractive targets. Humans cannot synthesize these amino acids, and they are instead obtained through the diet. Presumably, this means that targeting these bacterial amino acid synthesis pathways with antibiotics should be non-toxic for humans. The lysine biosynthesis pathway in particular is a well-known antibiotic target (Hutton et al., 2007; Triassi et al., 2014) because of its involvement in bacterial cell wall synthesis. In Gram-negative bacteria, *meso-*diaminopimelate (*meso*-DAP) is used as the crosslinker at the third position of a peptide stem that link individual peptidoglycan units to form the cell wall (Figure 1E); in Gram-positive bacteria, lysine serves this purpose (Hutton et al., 2007). Without the crosslinking of peptidoglycan, the cell wall loses stability and is unable to adequately protect and maintain separation of the internal and external environment, resulting in rupture. This critical role in peptidoglycan crosslinking and protein synthesis, as well as its lack of presence in animals (specifically humans), supports targeting the lysine biosynthetic pathways in the development of novel antibiotics.

Lysine biosynthesis occurs via one of two pathways: the alpha-aminoadipic (AAA) pathway is commonly found in yeast, fungi, and some archaea while the diaminopimelate (DAP) pathway is employed by photosynthetic organisms and most prokaryotes (Nishida et al., 1999; Velasco et al., 2002). DAP synthesis comprises three main steps: (1) conversion of aspartate to tetrahydrodipicolinate (THDP), (2) conversion of THDP to *meso*-diaminopimelate (*meso*-DAP), and (3) conversion of *meso*-DAP to lysine via an L,L-DAP intermediate (Figure 1). There are four known variants of the DAP pathway, distinguished by differences in the synthesis of *meso*-DAP: the two acyl pathways, the *meso*-diaminopimelate dehydrogenase (Ddh) pathway, and the L,L-diaminopimelate aminotransferase (DapL) pathway (Hudson et al., 2006). Notably, the DapL pathway variant utilizes DapL in a single transamination reaction to convert THDP directly to LL-DAP (Hudson et al., 2005, 2006).

DapL is a homodimer comprised of two subunits that are each ~50 kDa in size (Hudson et al., 2006), functionally active as a dimer with two active sites on a hinge. Each subunit is composed of a major and minor arm, where the major arm composes the dimer interface for each subunit. The DapL enzyme transfers an amino group plus a proton and electron pair from a donor molecule (in this case glutamate) to an acceptor molecule (THDP). Three key regions of the enzyme are involved in each aminotransferase reaction—one loop on the minor arm of the enzyme and two loops from the major arm in the dimer interface. Thus, each active site requires coordination between both subunits for optimal function (Velick and Vavra, 1962; Liepman and Olsen, 2004).

A number of organisms of extreme biotechnological and clinical relevance employ the DapL pathway, including phyla *Chlamydophila, Treponema, Leptospira*, and *Bacteroides* (Berry et al., 1987; Burstain et al., 1991; Finegold and Sussman, 2002; Wexler, 2007; Lindsay et al., 2010; Ansdell, 2012). Additionally, the DapL pathway is home to numerous commensal and environmental organisms with major biotechnological applications (Adams, 2019). Understanding essential metabolic pathways at a molecular level in these species is critical for future treatment development. For example, *Chlamydia trachomatis* is the causative agent of one of the most reported sexually transmitted diseases in the United States and the leading cause of infection-mediated blindness worldwide (Mishori et al., 2012). It is an obligate intracellular pathogen with high rates of lateral gene transfer between serotypes and largely uncharacterized genetic properties (Mabey, 2008). *Verrucomicrobium spinosum*, another organism that employs the DapL pathway, is the closest free-living relative to *C. trachomatis* (Griffiths and Gupta, 2007), an organism for which a model system would be advantageous to support research independent of cell culture and biological containment. *V. spinosum*, in place, has high potential for use as a safe experimental model in research and development of antimicrobial compounds targeting *C. trachomatis* and related pathogens.

Because of its presence in medically and biotechnologically relevant organisms, DapL is an attractive target for antibiotic, herbicide, and algaecide development. A previous comprehensive screening analysis identified compounds with antibiotic potential that inhibit DapL from *Arabidopsis thaliana, Leptospira interrogans, Chlamydomonas reinhardtii*, and *Verrucomicrobium spinosum* (McKinnie et al., 2014). Four of these compounds (rhodanine, barbiturate, hydrazide, and thiobarbiturate), all of which are derived from classes with different structural elements, specifically inhibit the activity of *V. spinosum* DapL (*Vs*DapL). However, DapL crystal structures from *L. interrogans, C. reinhardtii*, and *V. spinosum* are either not published or do not exist, and the binding conformation of the effective compounds are not experimentally determined. Unfortunately, this scenario reflects a common situation in research settings where inhibitory compounds are screened against potential targets with only structural information inferred from a related species, resulting in unknown docking positions.

Informatics resources have been utilized in recent years to explore structure-guided drug design and structure-activity relationships (SAR), even in cases without experimentally determined structural information and in cases before *in vitro* experimentation. This method often involves the use of molecular docking to identify putative binding sites (Abdolmaleki et al., 2017), molecular dynamics to supplement and refine such docking (Iqbal and Shah, 2018), and/or subsequent SAR studies to predict the biological activity of the compound based on similar structures (Fan et al., 2010). However, most previous studies are limited in the scope of the molecular dynamics simulations performed, the size of the simulations, or size of the molecule analyzed. Adding in the often modeled structures further confounds results and requires post-processing and analysis.

Here, a comprehensive, comparative molecular dynamics (MD) simulation package, DROIDS (Detecting Relative Outlier Impacts in Dynamic Simulations 2.0) (Babbitt et al., 2018), was used in conjunction with SWISS-MODEL (Pettersen et al., 2004; Biasini et al., 2014) and AutoDock Vina (Trott and Olson, 2010) to investigate the binding dynamics of the identified putative inhibitory lead compounds and *Vs*DapL inferred from homology modeling. DROIDS calculates the local modes of interaction between the residues, simulating inter- and intramolecular interactions, for two macromolecule sets over a time course for a defined number of replicates (Lewars, 2003; Jensen, 2007). It then compares the dynamics of the two macromolecules and presents an analysis of the differences in dynamic movement in the context of atomic fluctuations from the mean position. Here, we present putative docking positions of the four effective compounds tested and characterized via SAR and *in vitro* analyses in previous work (Fan et al., 2010) and provide investigative MD simulation data supporting the structural inference. The methods and results presented here not only address the efficacy of these tools in a common scenario of investigative antibiotic development but also can be applied and modified to both supplement and provide a rational guide in laboratory method development.

## Methods

### Multiple Sequence Alignment

Multiple sequence alignment was constructed using the Molecular Evolutionary Genetics Analysis (MEGA) (Kumar et al., 2016) tool with the DapL protein sequences from *V. spinosum* (NCBI Acc: WP_009961032.1)*, A. thaliana* (UniProt: Q93ZN9)*, C. trachomatis* (UniProt: G4NMX8), and *C. reinhardtii* (UniProt: A8IW39). Sequences were aligned via MUSCLE algorithm (Edgar, 2004). Conserved active site loops and residues were identified from the multiple sequence alignment, referencing those identified to interact with the natural ligand in the *A. thaliana* crystal structure and identified based on sequence homology between all four protein sequences.

### Homology Modeling of *Vs*DapL

Three dimensional homology model was constructed via SWISS-MODEL (Biasini et al., 2014; Waterhouse et al., 2018) after alignment to the template L,L-diaminopimelate aminotransferase from *C. reinhardtii* (PDB 3QGU) with 53.3% sequence identity. The template was chosen as the crystal structure with the best sequence identity to the enzyme based on a basic local alignment search tool (*BLAST*) search with the HHBlits algorithm (Remmert et al., 2011) against the SWISS-MODEL template library (Guex et al., 2009; Benkert et al., 2011; Biasini et al., 2014; Bertoni et al., 2017; Waterhouse et al., 2018). The model was built based on the alignment between reference and query protein sequence. Conserved coordinates were directly copied while insertions and deletions were modeled from the SWISS-MODEL fragment library.

### Structure Stabilization Testing

Structure stability and conformation testing was performed using the initial DROIDS pipeline development and prior to version 1 release. The solvated structures were each subjected to 1,000 cycles of minimization using a steepest-descent algorithm, followed by an 80-picosecond heating step where the temperature was gradually raised from 0 to 300K and then an 80-picosecond simulation allowing for the potential energy of the molecule to reach an equilibrium, followed by a 120-nanosecond sampling simulation. Atomic coordinates were calculated every 0.002 picoseconds and recorded every 0.2 picoseconds. Temperature and pressure were held constant for the equilibration and subsequent runs.

### Compound Structure Generation and Docking

Lead compounds (McKinnie et al., 2014) (Table 1) were exported in the simplified molecular-input line entry system format from the ChemBridge database and the highest quality, lowest energy three dimensional coordinates were generated with the CORINA server (Sadowski et al., 1994; Schwab, 2010).

**Table 1:** Compounds identified by McKinnie et al. (2014) and the corresponding structures.

**Compound**	**ChemBridge ID #**	**Chemical formula**	**Structure**
Hydrazide	5925714	C_17_ H_15_ N_3_ O_3_ S	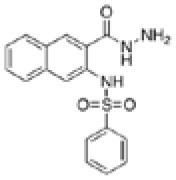
Rhodanine	6523070	C_19_ H_16_ Cl N O_2_ S_2_	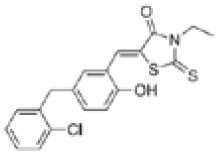
Barbiturate	6072466	C_22_ H_17_ N_3_ O_5_	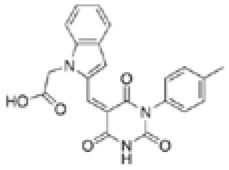
Thiobarbiturate	6088649	C_12_ H_13_ N_3_ O_2_ S	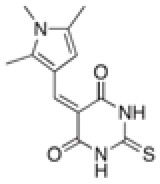

Three dimensional compounds were docked onto the DapL active site with the UCSF Chimera interface for Autodock Vina (Pettersen et al., 2004; Trott and Olson, 2010). The highest ranked conformation, with the best binding affinity reported, was chosen for each respective MD calculation set in DROIDS. For each run, the protein-ligand complex, the protein, and the ligand in its proper conformation identified by Vina were exported in PDB format. The resulting three files each were used.

### Molecular Dynamics Preparation

All chemical artifacts in the structures were removed manually prior to running DROIDS. A structure-based sequence alignment was constructed in UCSF Chimera for both chains on each set of structures to be compared (in this case an apo-DapL structure and DapL docked with a ligand) and saved in clustal format. Amber16 and the AmberTools packages were used in ligand and complex preparation to generate atomic coordinate files for MD calculations. The program pdb4amber was used to remove all extraneous water molecules and add hydrogen atoms prior to renumbering residues for downstream analyses. The antechamber package was used to estimate and parameterize the force fields surrounding the ligand using the general Amber force field (“GAFF”) simple harmonic function with AM1-BCC charge methods (Jakalian et al., 2000; Wang et al., 2004). Ligand coordinate files were generated from the antechamber ligand preparation. TeLEap was used to generate protein coordinate and topology files using the ff14SB protein force field parameters for both the apo-DapL and DapL-ligand complex (Maier et al., 2015). Atomic coordinates for both structures were utilized in subsequent MD calculations.

### Molecular Dynamics Sampling

The solvated structures were each subject to 1,000 cycles of minimization using a steepest-descent algorithm, followed by a 100-picosecond heating step to 300 K, followed by a 10-nanosecond equilibration step to allow for the potential energy of the molecule to reach an equilibrium before 300 production sampling runs of 0.5 nanoseconds. Starting coordinates were randomly generated by the DROIDS software at the onset of each sampling run. Temperature and pressure were held constant for the equilibration and subsequent runs.

### Molecular Dynamics Post-processing

The CPPTRAJ package provided in Ambertools was used to generate trajectories for each residue and calculate Root Mean Square Deviation (RMSD) at an amino acid resolution. The calculated atom fluctuation profiles were used to calculate signed symmetric Kullback–Leibler (KL) divergences in local atom fluctuation distributions from each amino acid on the polypeptide backbone, and *P*-values from a Benjamini–Hochberg corrected Kolmogorov-Smirnov (KS) test. All post-processing analyses were performed in R and presented in graphical representation. UCSF Chimera was used for structure depiction.

## Results

### Key Amino Acids of Known DapL Active Sites Are Conserved in *V. spinosum* DapL

**Figure 2:**
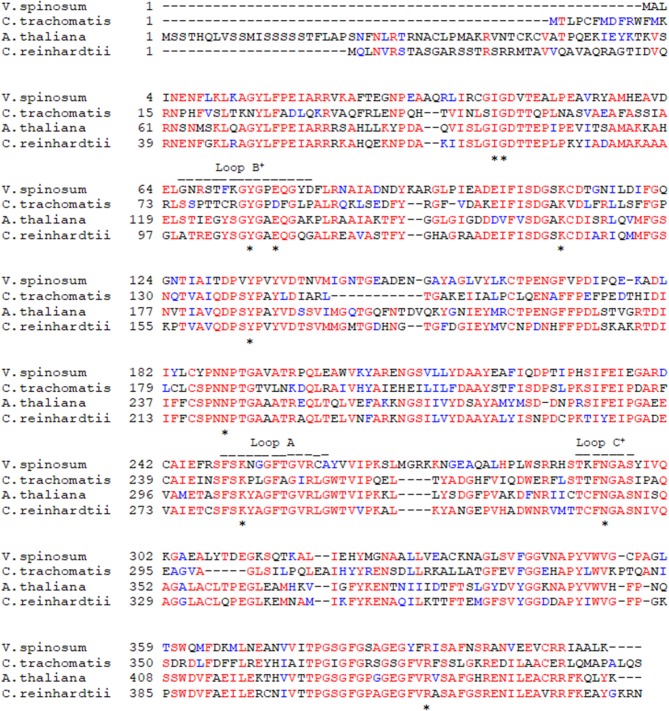
Multiple protein sequence alignment of the *Vs*DapL to reference sequences. *Conserved active site residues; − − −, residues in loops lining the active site, and + denotes contribution from the opposing chain.

**Figure 3:**
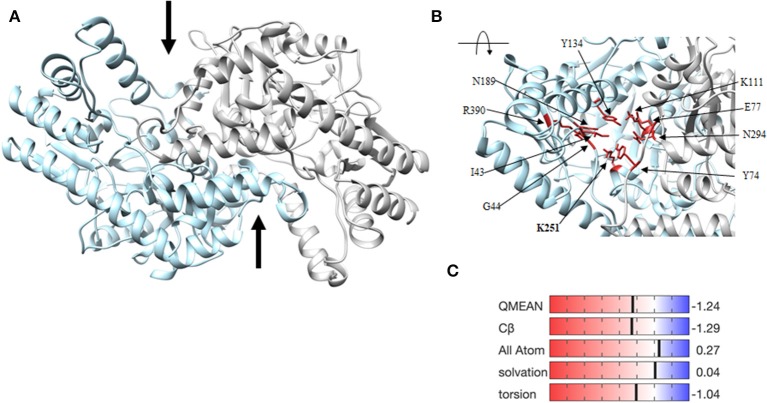
Homology model of the *Vs*DapL enzyme. **(A)** Three dimensional ribbon model depicting the predicted structure, a homodimer (gray and blue). The two black arrows denote the predicted active sites. **(B)** Ribbon model of the rotated, centered *Vs*DapL active site showing active site residues (red) identified from multiple sequence alignment. **(C)** Z-score data when the model is compared to high quality X-ray crystal structures of similar size.

To identify key active site amino acid residues in the DapL ortholog from *V. spinosum* (*Vs*DapL), we constructed a multiple sequence alignment using the sequences of DapL orthologs where structures have already been experimentally determined [*V. spinosum* (PDB: WP_09961032.1), *A. thaliana* (UniProt: Q93ZN9), C. trachomatis (UniProt: G4NMX8), and C. reinhardtii (UniProt: A8IW39)]. The key residues in the active site were highly conserved across all organisms. Loops that line the active site in *V. spinosum* were predicted to reside between F249 and A261 (Loop A), as well as those from the opposing chain between residues G66 and D81 (Loop B), and T291 and S297 (Loop C). In addition, key conserved active site residues were identified as I43, G44, Y74, E77, K111, Y134, N189, K251, N294, R390 (Figures 2, 3B) (McKinnie et al., 2014).

The three-dimensional homology model predicts a homodimer with two v-shaped clefts that correspond to the putative active sites (Figure 3A) and the active sites agree with the alignment findings above. The model quality, assessed with z-scores for the QMEAN function (−1.24), all atom pairwise energy (0.27), CBeta interaction energy (−1.29), solvation energy (0.04), and torsion angle energy (−1.04), indicates an acceptable structure prediction (Figure 3C).

### DapL Is More Stable *in silico* as a Homodimer

**Figure 4:**
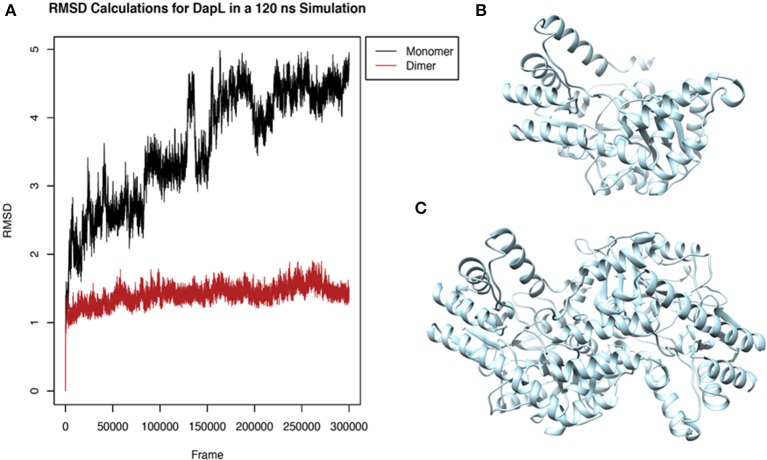
Stability of *Vs*DapL comparing monomer and dimer. **(A)** Root Mean Square Deviation (RMSD) values for the fluctuation of the alpha-carbons of each amino acid from the mean position of the backbone calculated for the molecular dynamics sampling runs of the **(B)** *Vs*DapL monomer and **(C)** *Vs* DapL homodimer.

To determine structural stability, RMSD calculations of the fluctuation of the alpha-carbons of each amino acid from their mean positions were used to compare the homodimer and monomer forms of *Vs*DapL. As a monomer, the early sampling of *Vs*DapL coordinates generate a RMSD value of 2 angstroms. Over time, however, the RMSD increases and nears 5 angstroms. On the contrary, the *Vs*DapL homodimer stabilizes just above a RMSD of 1 angstrom for the duration of the sampling time (Figure 4). The higher and consistently rising RMSD values resulting from the monomer dynamics indicate more instability in the system, while the constant lower RMSD values from the dimer indicate a much more stable structure. Overall, the dimer settled into a lowest-energy conformation and maintained that general pattern for the duration of the simulation, as opposed to a constantly fluctuating set of conformations in the monomer. This is also consistent with previous findings, where other DapL orthologs are homodimers in solution.

### Rhodanine, Barbiturate, Hydrazide, and Thiobarbiturate Association With *Vs*DapL Is Supported by Molecular Dynamics Simulations

**Figure 5:**
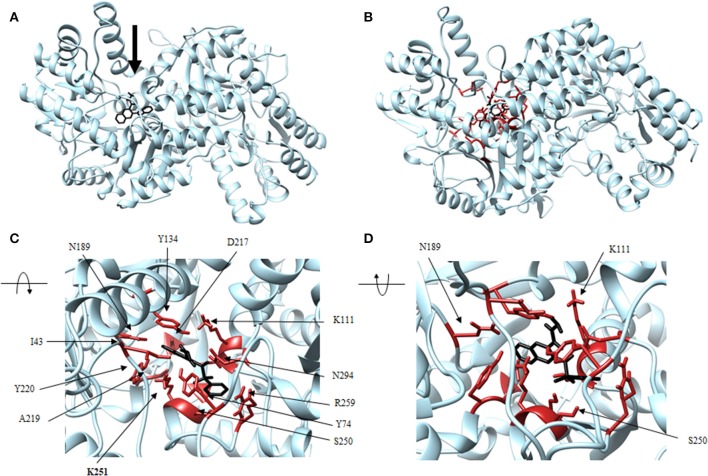
Structure of hydrazide (black) associated with *Vs*DapL (blue). **(A)** Ribbon conformation showing binding pocket (black arrow). **(B)** Ribbon conformation showing interacting residues (red). **(C)** Rotated, centered binding pocket showing interacting residues (red, labeled). **(D)** Rotated, centered binding pocket, denoted by arrow, with two labeled interacting residues (red) for orientation.

**Figure 6:**
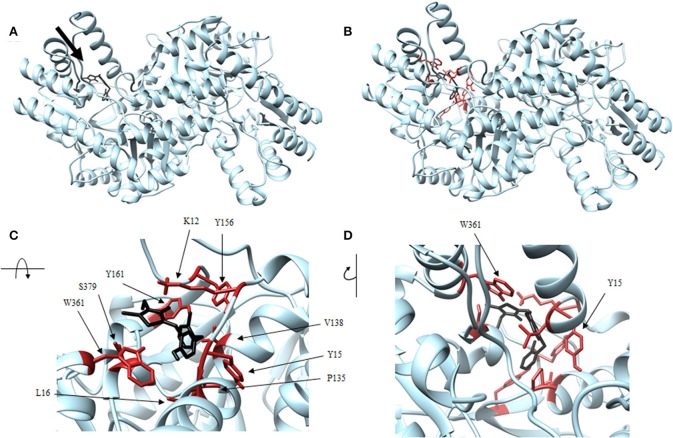
Structure of rhodanine (black) associated with *Vs*DapL (blue). **(A)** Ribbon conformation showing binding pocket (black arrow). **(B)** Ribbon conformation showing interacting residues (red). **(C)** Rotated, centered binding pocket showing interacting residues (red, labeled). **(D)** Rotated, centered binding pocket, denoted by arrow, with two labeled interacting residues (red) for orientation.

**Figure 7:**
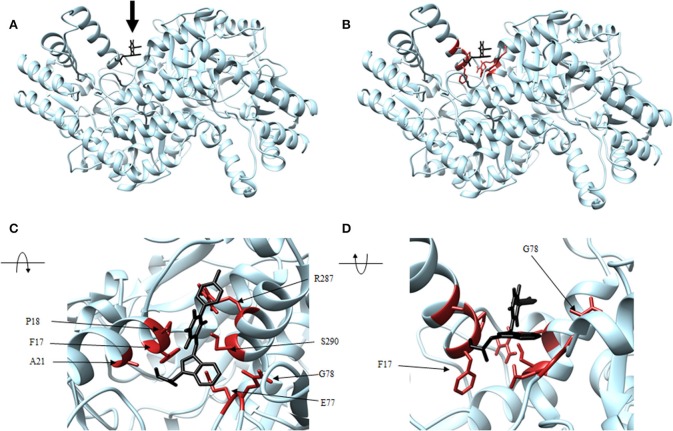
Structure of barbiturate (black) associated with *Vs*DapL (blue). **(A)** Ribbon conformation showing binding pocket (black arrow). **(B)** Ribbon conformation showing interacting residues (red). **(C)** Rotated, centered binding pocket showing interacting residues (red, labeled). **(D)** Rotated, centered binding pocket, denoted by arrow, with two labeled interacting residues (red) for orientation.

**Figure 8:**
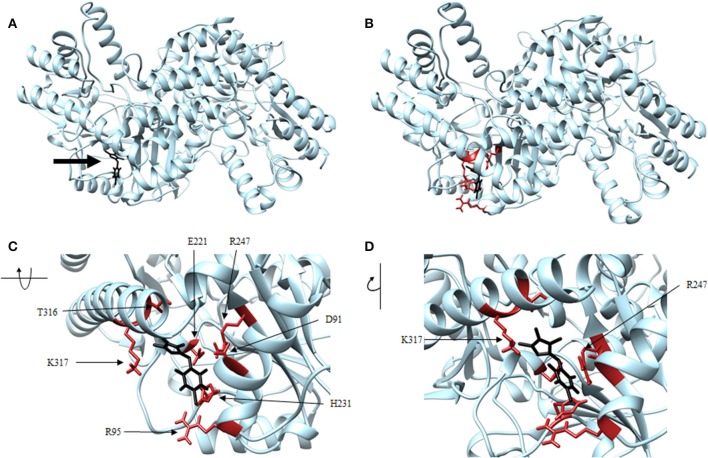
Structure of thiobarbiturate (black) associated with *Vs*DapL (blue). **(A)** Ribbon conformation showing binding pocket (black arrow). **(B)** Ribbon conformation showing interacting residues (red). **(C)** Rotated, centered binding pocket showing interacting residues (red, labeled). **(D)** Rotated, centered binding pocket, denoted by arrow, with two labeled interacting residues (red) for orientation.

Four antagonist lead compounds (rhodanine, barbiturate, hydrazide, and thiobarbiturate) were docked into the *Vs*DapL homology model to represent modeling the molecular dynamics of macromolecular complexes without actual structural data. All antagonistic compounds successfully associated with the *Vs*DapL active site with a potential energy lower than the enzyme in its native state and with all potential energies at the same order of magnitude, but with varying degrees of affinity (−6.5 to −7.6 kcal/mol, data not shown) as indicated by the RMSD values when compared to the best identified conformation. The best docking position in the structure also varied for each compound (Table 2). The hydrazide molecule bound close to the active site (Figure 5), the rhodanine molecule docked in the minor arm of one subunit (Figure 6), the barbiturate molecule docked on the outer edge of the structure near an active site (Figure 7), and the thiobarbiturate molecule docked on the outer surface of the protein (Figure 8).

**Table 2:** The binding affinity for and residues that interact with each identified compound used in this study.

**Compound**	**Binding affinity (Kd) (kcal/mol)**	**Interacting residues**
Hydrazide	−7.6	43^*^, 74^*^, 111^*^, 110, 134^*^, 189^*^, 217, 250, 251^*^^*^, 259, 294^*^
Rhodanine	−8.3	12, 15, 16, 135, 138, 156, 161, 361, 379
Barbiturate	−7.6	17, 18, 21, 77^*^, 78, 287, 290
Thiobarbiturate	−6.4	91, 95, 221, 231, 247, 316, 317

* Denotes residues that are associated with the active site. **Denotes catalytic residue.

In addition, identified contacts support that the four small molecule antagonists bound to different locations on DapL. Hydrazide bound to the majority of residues in the active site, including the catalytic lysine (K251). Rhodanine and thiobarbiturate did not bind to any residues that compose active site, and barbiturate bound near some active site residues but not directly in the active site (Table 2).

**Figure 9:**
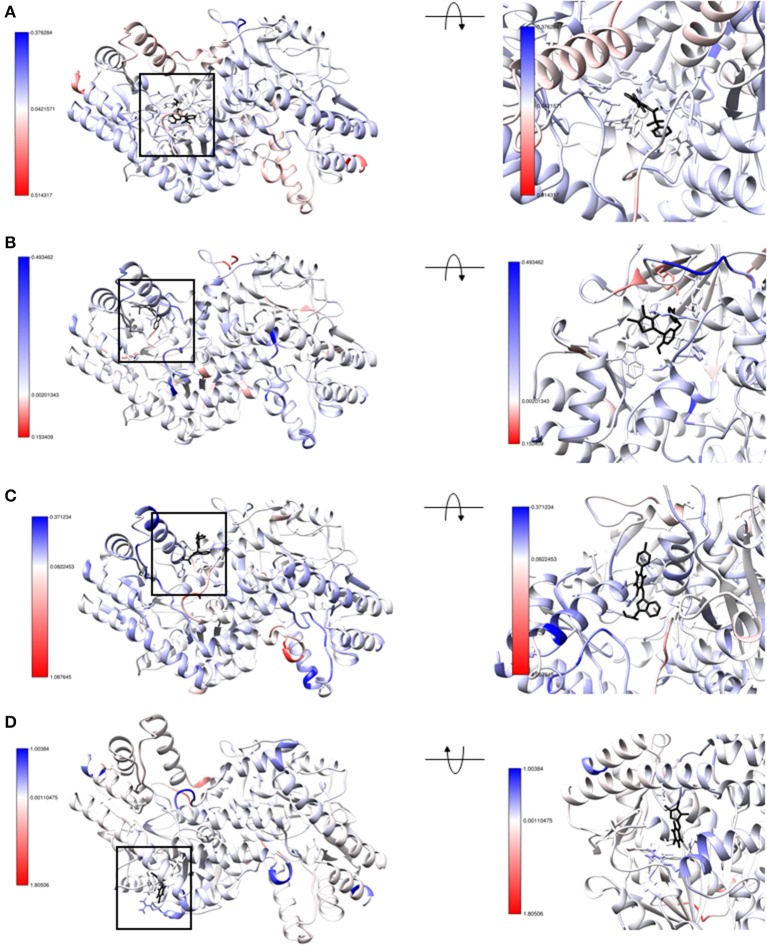
Structure of **(A)** hydrazide **(B)** rhodanine **(C)** barbiturate and **(D)** thiobarbiturate associated with *Vs*DapL colored by signed symmetric KL divergences in local atom fluctuation distributions of each amino acid on the polypeptide backbone. The small molecule inhibitors are colored in black. The KL divergences shown are color-mapped to temperature on the protein with red indicating softer regions with amplified fluctuation and blue indicating stabilized regions with dampened fluctuation. The magnitude of divergence is shown in the color key beside the structure. (left) front-facing view with a black box to direct attention to the region (right) centered and rotated to show the interacting residues.

Molecular dynamics of each *Vs*DapL-antagonist macromolecular complex was compared to the unbound *Vs*DapL molecule in terms of differences in fluctuations for 500 replicate simulations with random initialization coordinates. The differences in fluctuation were then color-mapped to *Vs*DapL at an amino-acid resolution (Figure 9). Differences in fluctuation were found between the unbound molecule and bound complex for all four cases. The dynamics of most, if not all, *Vs*DapL residues that interacted with the compound in the pocket were stabilized upon association. In only two instances (Figures 9B,C) were the dynamics of a single residue amplified upon compound association. However, both residues are near loops and on the exterior of the protein, so some instability would be expected even upon settling into a bound conformation. Additionally, the color-mapping of the true *Vs*DapL active sites show stabilization upon compound association. This color mapping is supported by plots of the average fluctuation of each residue from the mean position of both bound and unbound structures, the signed KL divergence of the fluctuations, and significance based on a KS test (Supplemental Figure 1). Overall, the local stabilization upon compound association indicates the binding positions identified with the docking software are accurate.

## Discussion

The *Vs*DapL homodimer is more stable than the monomer, and DapL is naturally crystallized as a homodimer. Based on the sample size and volume of data collected, combined with experimentally determined crystal structures of multiple DapL enzymes, we must emphasize that all DapL computational analyses (docking, molecular dynamics, etc.) should involve the homodimer. Conclusions drawn from calculations on one subunit are not biologically accurate. Because DROIDS is equipped to perform dynamics calculations on large, multi-subunit proteins and map the statistics at an amino acid resolution, it provided us with a unique opportunity for DapL and other dimer dynamic analyses.

All antagonistic compounds in this study have the potential to interact with DapL *in vivo*, a conclusion supported by previous work (Fan et al., 2010; McKinnie et al., 2014). In our analyses, compounds were only docked into one active site because of the conformational change associated with the DapL mechanism of action. However, because all DapL structures characterized to date are indeed homodimers, we can speculate that the inhibitory lead compounds have the capacity to associate in at least two sites. In fact, a test docking of two rhodanine molecules onto DapL indicates better binding affinity for both together than the single molecule. A kinetic analysis of the docking pattern of these molecules would provide additional support for this hypothesis. However, docking protocols are not as robust as molecular dynamics simulations, nor do they flawlessly represent biological conditions.

Additionally, molecular dynamics simulations require precise and refined calculations that take into account extremely complex physical characteristics, and as a caveat they must be cautiously analyzed for robustness and consistency. For example, molecules must be in their lowest potential energy wells for consistent and meaningful calculations. This task becomes exponentially more difficult as the size of the molecule increases. If the atomic coordinates are calculated for longer duration, or sampled for a different number of replicates, it is possible that the refined, amino-acid resolution results would differ. However, the trends reported from our simulations, of stabilization in both the binding pocket and more loosely at the true active site of the enzyme, provide strong support for the putative binding positions of the compounds. Because the inhibition potential of the four small molecules was previously determined, we were able to draw conclusions based on the consistency in the results of the molecular dynamics simulations. Indeed, all simulations supported the previously drawn experimental conclusions. If the biological effect of these compounds was not known, SAR analyses could be performed with the top compounds identified in the docking and molecular dynamics screening. This would provide additional insights as to the potential biological effect of the compounds and the effect of modifications to these compounds to further guide future work. The efficacy of this study provides rationale for using any iteration of this work (modeling, docking, or molecular dynamics) as a springboard to suggest future experimental work in the rational design of experimental antibiotic compounds.

In conclusion, this and previous studies have shown that DapL is a potential target for the development of narrow spectrum antibiotics, and *Vs*DapL has high potential for the development of a model system for DapL from related pathogenic organisms. Additionally, this study supports the great potential scope of the application of molecular dynamics to biological molecules to infer function, form, and effect of interactions in situations where the crystal structure may not be known. The success of this exploratory study in identifying the putative binding locations of four inhibitory compounds only touches the surface of the capabilities of molecular dynamics software. If applied correctly, molecular dynamics software can provide direction for major questions in the biological sciences across disciplines.

## Data Availability Statement

The datasets generated for this study are available on request to the corresponding author.

## Author Contributions

This work was conceived by AH and LA. The experiments were conducted by LA, PR, and JM. AH, LA, GB, RN, and RD wrote the manuscript.

### Conflict of Interest

The authors declare that the research was conducted in the absence of any commercial or financial relationships that could be construed as a potential conflict of interest.

## Supplementary Material

Supplemental Figure 1 Average atom fluctuation profiles (top plot), signed symmetric KL divergences in local atom fluctuation distributions of each amino acid on the polypeptide backbone (middle plot), and *P*-values from a Benjamini–Hochberg corrected KS test indicating significant differences in dynamics is also shown (bottom plot) for **(A)** hydrazide **(B)** rhodanine **(C)** barbiturate and **(D)** thiobarbiturate.
