# Supplementary figures and images for: Comparative Molecular Dynamics Simulations Provide Insight Into Antibiotic Interactions: A Case Study Using the Enzyme L,L-Diaminopimelate Aminotransferase (DapL)
Source: Front Mol Biosci. 2020 Mar 24;7:46. doi: 10.3389/fmolb.2020.00046 (PMC7113581; doi:10.3389/fmolb.2020.00046)

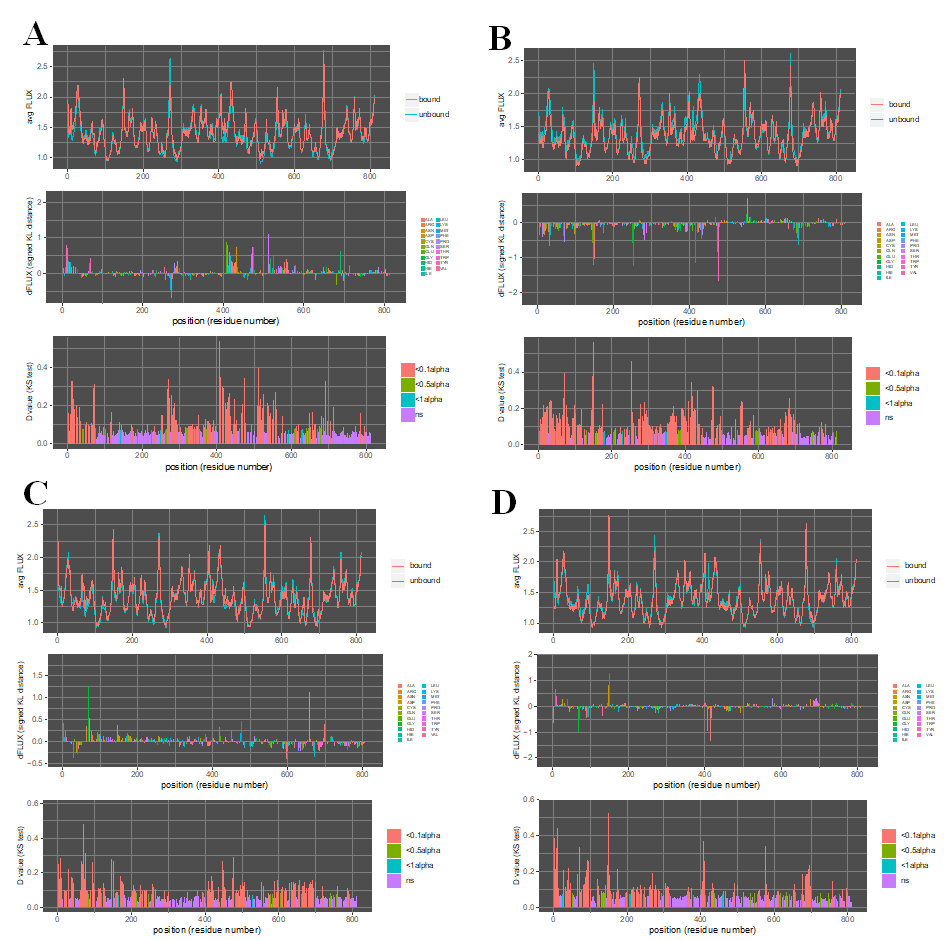

Supplement: Supplemental Figure 1 — Average atom fluctuation profiles (top plot), signed symmetric KL divergences in local atom fluctuation distributions of each amino acid on the polypeptide backbone (middle plot), and P-values from a Benjamini–Hochberg corrected KS test indicating significant differences in dynamics is also shown (bottom plot) for (A) hydrazide (B) rhodanine (C) barbiturate and (D) thiobarbiturate. [file Image_1.TIF]
